# [Rh(L-alaninate)(1,5-Cyclooctadiene)] Catalyzed Helix-Sense-Selective Polymerizations of Achiral Phenylacetylenes

**DOI:** 10.3390/polym10111223

**Published:** 2018-11-03

**Authors:** Qingyu Wang, Hongge Jia, Yongqiang Shi, Liqun Ma, Guoxing Yang, Yazhen Wang, Shuangping Xu, Jianjun Wang, Yu Zang, Toshiki Aoki

**Affiliations:** 1 College of Materials Science and Engineering, College of Chemistry and Chemical Engineering, Heilongjiang Province Key Laboratory of Polymeric Composition Material, Qiqihar University, Qiqihar 161006, China; wqy199323@163.com (Q.W.); maliqun6166@163.com (L.M.); wyz6166@163.com (Y.W.); xshp_1979_1999@163.com (S.X.); wangjianjun860505@163.com (J.W.); zangyu25@163.com (Y.Z.); toshaoki@eng.niigata-u.ac.jp (T.A.); 2 Department of Materials Science and Engineering and The Shenzhen Key Laboratory for Printed Organic Electronics, South University of Science and Technology of China, Shenzhen 518055, China; 3 Daqing Petrochemical Research Center, Petrochemical Research Institute, China National Petroleum Corporation, Daqing 163714, China; ygx459@petrochina.com.cn; 4 Graduate School of Science and Technology, Niigata University, Niigata 950-2181, Japan

**Keywords:** helix-sense-selective polymerization, phenylacetylene, chiral rhodium catalyst, L-alaninate

## Abstract

The [Rh(L-alaninate)(cod)] (cod = 1,5-Cyclooctadiene) complex was synthesized and characterized. Asymmetric polymerizations of achiral phenylacetylene with two hydroxyl groups and a dodecyl group (**DoDHPA**) were performed by using the rhodium complex as the catalyst to provide polymers with a higher molecular weight (>10^5^) than the polymers obtained using the [Rh(cod)Cl]_2_ initiator systems. The resulting polymers showed circular dichroism (CD) signals at approximately 310 and 470 nm, indicating that they have a preferential one-handed helical structure. The helix sense in the polymer main chain was controlled by the sign of the catalyst chirality. These findings suggest that the rhodium complex with a chiral amine is the true active species for the helix-sense-selective polymerization of **DoDHPA**. The [Rh(L-alaninate)(cod)] complex also exhibits high catalytic activity in the polymerization of phenylacetylene (**PA**) to give a high yield and molecular weight. All these results demonstrate that this Rh complex is an excellent catalyst for the polymerization of phenylacetylene monomers.

## 1. Introduction

Asymmetric catalysis is one of the best methods for the synthesis of optically active organic compounds [1,2]. The design and development of novel catalytic systems for asymmetric synthesis and polymerizations have been active research areas in recent years [3,4,5,6,7,8,9,10]. In particular, in the field of phenylacetylene (**PA**) asymmetric polymerizations, Rh catalysts have been found to play a vital role and cannot be replaced by other metal catalysts [11].

T. Aoki et al. [12] polymerized p-dodecyloxy-m,m-dihydroxyphenylacetylene (**DoDHPA**) by [Rh(nbd)Cl]_2_/(S)-PEA (here, nbd is 2,5-norbornadiene and PEA is 1-phenylethylamine) to produce the corresponding polymer that exhibited large cotton effects in the circular dichroism (CD) spectra. The polymerization was named helix-sense-selective polymerization (**HSSP**). In this **HSSP**, the chirality sign of the formed main chain was controlled by the chirality (R) or (S) of the PEA cocatalyst. The authors speculated that (R) or (S)-PEA coordinates to the rhodium catalyst, and the formed complex may be a chiral catalytic center in **HSSP** [12,13]. Self-helix-sense-selective polymerization (**SHSSP**) was also carried out for phenylacetylenes with an L-amino ether residue and two hydroxy groups (**ORDHPAs**: **OADHPA**, **OVDHPA**, and **OPDHPA**) [13] (Chart S1, Supplementary Materials) by using the achiral [Rh(nbd)Cl]_2_ catalytic system. The chiral amino ether residues in the monomer and the resulting polymers were thought to act as the ligand and cocatalyst of **HSSP** instead of (R)-and (S)-PEA. However, the mechanism of **SHSSP** was not proven directly. Therefore, isolation of the real active species of **HSSP** or **SHSSP** was desired but was difficult due to the low stability of the Rh-amine complex [14,15].

T. Masuda and coworkers have designed and developed Rh catalyst systems for the polymerizations of substituted acetylenes in the past several years [7,9]. Two types of catalysts, namely, [Rh(nbd)Cl]_2_ and (nbd)Rh^+^[η^6^-(C_6_H_5_)B^-^(C_6_H_5_)_3_], were synthesized; here, the diene ligands were commonly 2,5-norbornadiene (nbd), 1,5-cyclooctadiene (cod), endo-dicyclopentadiene (dcp), tetrafluorobenzobarrelene (tfb), and tetrachlorobenzobarrelene (tcb). The complexes (tfb)Rh^+^[η^6^-(C_6_H_5_)B^-^(C_6_H_5_)_3_] and (tcb)Rh^+^[η^6^-(C_6_H_5_)B^-^(C_6_H_5_)_3_] can induce helix-sense-selective polymerization of **DoDHPA**, but these catalysts were not crystallized from the reaction mixture [7]. M. A. Casado polymerized **PA** by using a rhodium complex with mixed anionic N–O bidentate ligands. However, the catalyst was not isolated from the ternary systems [10]. The rhodium complex of tfb ([Rh(tfb)Cl]_2_) is more catalytically active than the corresponding nbd complex in the polymerization of terminal alkynes ([Rh(nbd)Cl]_2_). However, chiral ligands have been developed for the transition metals that have displayed high activity and enantioselectivity in catalytic asymmetric reactions [11,14].

**Scheme 1:**
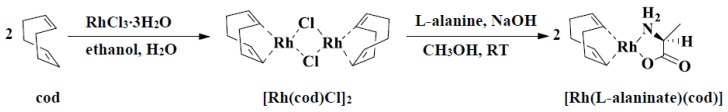
Synthetic route to catalyst [Rh(L-alaninate)(cod)].

In this paper, we report for the first time the crystal chiral [Rh(L-alaninate)(cod)] (cod = 1,5-Cyclooctadiene) catalyst (see Scheme 1) that is very easily obtained and is suitable for the asymmetric polymerization of **DoDHPA**. The catalytic efficiency for **DoDHPA** and **PA** was found to be very high.

## 2. Experimental Section

All solvents were dried using standard methods and were distilled over CaH_2_ under reduced pressure prior to use. Commercially available compounds were used without further purification. RhCl_3_·3H_2_O and all other chemicals products including the L-amino acids and **PA** were purchased from Shanghai Darui Finechem Co., Ltd. (Darui, Shanghai, China).

According to Scheme 1, the [Rh(L-alaninate)(cod)] complex was synthesized and isolated; the following reaction procedures were conducted under dry nitrogen.

**Figure 1:**
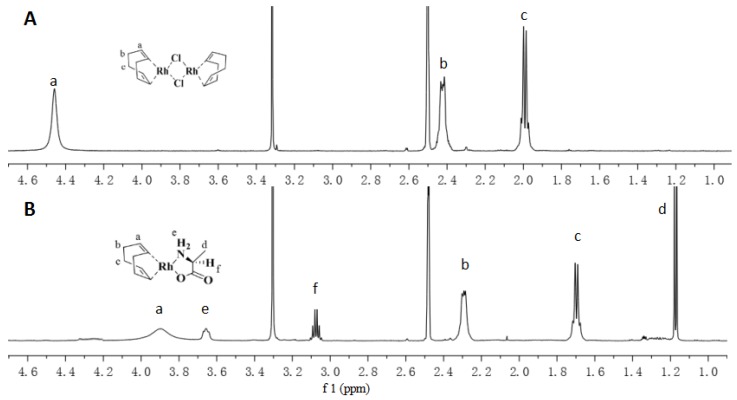
(**A**) ^1^H NMR spectra of [Rh(cod)Cl]_2_ and (**B**) [Rh(L-alaninate)(cod)] in DMSO-*d*_6_ at 25 °C.

[Rh(cod)Cl]_2_: The compound was synthesized and purified according to the procedure reported in the literature (see Figure 1A; Figures S1 and S2, Supplementary Materials) [16].

[Rh(L-alaninate)(cod)]: A solution of L-alanine (13.20 mg, 0.130 mmol) and NaOH (5.50 mg, 0.130 mmol) in H_2_O (0.60 mL) was added to a yellow suspension of [Rh(cod)Cl]_2_ (30.00 mg, 0.06 mmol) in methanol (2.00 mL). The obtained clear yellow solution was stirred for 1 h at room temperature. The mixture was cooled at 5 °C until precipitation out from methanol, and then the precipitate was isolated by centrifugation. The product was obtained as a yellow powder (30.00 mg, 0.100 mmol, 77.9%). ^1^H NMR (600 MHz, DMSO-*d*_6_, TMS, δ): 3.89 (br, 4H, CH=CH), 3.66 (br, 2H, CH_3_CHNH*_2_*), 3.07 (m, 1H, CH_3_CHNH_2_), 2.29 (m, 4H, CH_2_CH_2_), 1.67–1.71 (d, 4H, CH_2_CH_2_), 1.16 (d, 3H, CH_3_) (see Figure 1B). ^13^C NMR (150 MHz, DMSO-*d*_6_, TMS, δ): 183.70 (*C*OO), 129.04 (*C*H=CH), 52.93 (CH_3_*C*H), 30.60 (*C*H_2_CH_2_), 20.92 (*C*H_3_CH) (Figure S3, Supplementary Materials). IR (KBr): 3444.85, 3257.97, 2935.18, 2877.26, 2830.19, 1633.71, 1572.32, 1382.10, 1339.62, 1284.56, 1186.59, 1112.61, 963.04, 856.67 cm^−1^ (Figure S4, Supplementary Materials). Elemental analyses for C_11_H_1__8_NO_2_Rh: calc., %: C 44.16, H 6.02, N 4.68; found, %: C 44.73, H 5.13, N 4.82.

[Rh(L-alaninate)(nbd)]: A solution of L-alanine (12.20 mg, 0.130 mmol) and NaOH (5.20 mg, 0.130 mmol) in H_2_O (0.60 mL) was added to a yellow suspension of [Rh(nbd)Cl]_2_ (35.40 mg, 0.070 mmol) in methanol (2.00 mL). The obtained clear yellow solution was stirred for 1 h at room temperature. The mixture was cooled at 5 °C until precipitation out from methanol, and then the precipitate was isolated by centrifugation. The product was obtained as a light yellow powder (18.85 mg, 0.070 mmol, 39.6%) (Scheme S1, Supplementary Materials). ^1^H NMR (600 MHz, DMSO-*d_6_*, TMS, δ): 4.03 (m, 4H, CHCH=CHCH), 3.61 (s, 2H, CH_3_CHN*H_2_*), 2.40 (m, 1H, CH_3_CHNH_2_), 1.98 (s, 2H, CHCH_2_CH), 1.17 (t, 2H, CHCH_2_CH), 0.92 (t, 3H, CH*_3_*CHNH_2_) (Figure S5, Supplementary Materials). IR (KBr): 3427.41, 2923.77, 2857.82, 1618.95, 1452.57, 1382.12, 1286.94, 1196.25, 1080.09, 999.89, 798.29 cm^−1^ (Figure S6, Supplementary Materials). Elemental analyses for C_10_H_14_NO_2_Rh: calc., %: C 42.42, H 4.95, N 4.95; found, %: C 45.87, H 6.03, N 4.52.

The achiral monomer **DoDHPA** was synthesized as described previously [12]. Yield 46.0%. ^1^H-NMR (600 MHz, CDCl_3_, TMS, δ): 7.49 (s, 2H, PhH), 4.70 (s, 4H, CH_2_OH), 3.88 (t, 2H, OCH_2_CH_2_), 3.05 (s, 1H, C≡CH), 1.93 (br, 2H, CH_2_O*H*), 1.78 (m, 2H, OCH_2_CH_2_CH_2_), 1.26-1.48 (br, 18H, OC_2_H_4_(CH_2_)_9_CH_3_), 0.88 (t, 3H, CH_3_) (Figure S7, Supplementary Materials). IR(KBr): 3356.96, 3296.25, 2918.52, 2853.32, 1463.06, 1357.39, 1211.24, 1140.79, 1055.35, 1014.88, 898.72, 803.53, 717.34, 657.39, 611.67 cm^−1^ (Figure S8, Supplementary Materials).

Polymerization: **DoDHPA** and **PA** were polymerized by using [Rh(L-alaninate)(cod)] and [Rh(cod)Cl]_2_. A typical polymerization procedure was as follows: a solution (0.20 mL) of [Rh(L-alaninate)(cod)] (0.17 mg, 0.577 μmol) in dry toluene was added to a dry toluene solution (0.40 mL) of **DoDHPA** (20.00 mg, 57.700 μmol). Polymerization was performed at 35 °C for 24 h. The formed polymer was isolated by precipitation in a large amount of methanol, centrifuged and dried under vacuum to a constant weight. ^1^H NMR (600 MHz, 1,2-dichlorobenzene-*d*_4_, TMS, δ): 7.33 (s, 2H, PhH), 6.18-5.63 (m, 1H, PhC=CH), 4.95 (s, 4H, C*H*_2_OH), 4.06 (t, 2H, OCH_2_CH_2_), 1.77 (t, 2H, CH_2_OH), 1.59 (s, 2H, OCH_2_CH_2_CH_2_), 1.11-1.23 (m, 18H, OC_2_H_4_(CH_2_)_9_CH_3_), 0.53 (t, 3H, CH_3_) (Figure S9, Supplementary Materials). IR (KBr): 3351.22, 2924.05, 2853.28, 1603.86, 1464.87, 1382.03, 1352.11, 1299.25, 1211.92, 1150.04, 1069.08, 1042.90, 986.98, 913.62, 835.98 cm^−1^ (Figure S10, Supplementary Materials).

## 3. Instruments

The number and weight-averaged molecular weights (*M*_n_ and *M*_w_, respectively) and dispersity (*M*_w_/*M*_n_) of the polymers were measured by gel permeation chromatography (GPC) (Polymer Laboratories, United Kingdom) at 40 °C. A nuclear magnetic resonance (Mode, Bruker, Germany) instrument (^1^H NMR and ^13^C NMR) was used to determine the molecular structure of the monomer and the catalysts. Infrared spectra were recorded using an IR-spectrum One B (PE, Austin, TX, USA) instrument. CD spectra were recorded using a MOS-450 (Bio-Logic, Seyssinet-Pariset, France) instrument. SEM images were recorded using an S-4300 (Hitachi, Tokyo, Japan) apparatus. XRD spectra were recorded using a D8-FOCUS (Bruker, Karlsruhe, Germany) instrument. Mass spectra were recorded using an UltiMate 3000 UPLC/Q-Exactive Orbitrap MS (Thermo, Hampton, NH, USA) instrument.

## 4. Results and Discussion

In this work, the rhodium catalyst [Rh(L-alaninate)(cod)] was purified using the recrystallization method [17,18,19,20]. The isolated [Rh(L-alaninate)(cod)] complex was a yellow powder, that was crystallized from dichloromethane. The catalyst has a polygonal shape, and the crystal surface is very smooth (Figure S11, Supplementary Materials). The XRD spectrum showed good crystallizing effect (Figure S12, Supplementary Materials). The mass spectrum showed that the peak at 299 corresponds to the formation of [Rh(L-alaninate)(cod)] (Figure S13, Supplementary Materials). The [Rh(L-alaninate)(nbd)] complex achieved similar results with [Rh(L-alaninate)(cod)] (Figures S14 and S15, Supplementary Materials). We concluded that the crystals of the catalysts were obtained by the recrystallization.

The ^1^H-NMR spectrum (Figure 1) of the [Rh(L-alaninate)(cod)] dissolved in DMSO-*d*_6_ was obtained. The peaks of (–CH_3_), amino group (–NH) and methyne (–CH) in L-alaninate appeared separately at 1.16–1.17 ppm, 3.66 ppm and 3.07–3.08 ppm. The coordination of L-alanine to rhodium is supported. When L-alanine was coordinated to rhodium, the chemical shift of the olefinic and methylene protons in cod changed from 4.45, 2.43, and 1.99 ppm (Figure 1A) to 3.89, 2.29, and 1.69 ppm (Figure 1B), respectively [6]. The coordination of cod to rhodium is also supported. This chemical shift change arises because L-alanine provides an easily accessible coordination vacancy through a potential effect on the metal center [21]. On the basis of these ^1^H-NMR spectra as well as the results of the ^13^C NMR, IR, and elemental analyses, it was concluded that the novel chiral rhodium catalyst containing cod and L-alanine ([Rh(L-alaninate)(cod)]) was synthesized successfully.

**Scheme 2:**
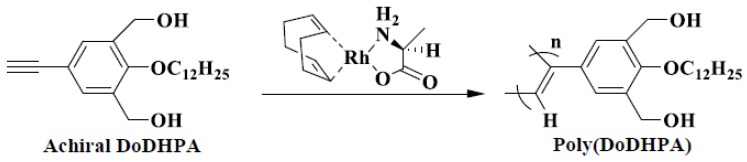
Helix-sense-selective polymerization of **DoDHPA** by using [Rh(L-alaninate)(cod)] as catalyst.

Here, we report the asymmetric polymerization of achiral phenylacetylene monomer (**DoDHPA**) by using the crystalline chiral Rh catalyst, as shown in Scheme 2.

The achiral monomer (**DoDHPA**) was polymerized by using the binary [Rh(cod)Cl]_2_/(chiral amine) catalytic system, with [Rh(cod)Cl]_2_ as the main catalyst and L-prolinol, L-valinol, L-alaninol, or (S)-PEA as cocatalysts, to produce polymers in low yield (8%–31%) with moderate *M*_w_ 2.93 × 10^4^–28.50 × 10^4^ (Table 1, entries 1–4). Similar results were also reported by Trhlikova and Mawatari [22,23]. To obtain polymers with higher yield and *M*_w_ values, we attempted to prepare a novel high-efficiency catalyst [Rh(L-alaninate)(cod)]. We polymerized **DoDHPA** using the isolated [Rh(L-alaninate)(cod)] catalyst without a cocatalyst to lead a high yield (72%) and large molecular weight (55.43 × 10^4^), as presented in Table 1. Similar results were obtained when a binary [Rh(nbd)Cl]_2_/(chiral amine) catalytic system and novel isolated [Rh(L-alaninate)(nbd)] catalyst without a cocatalyst were used to polymerize **DoDHPA**. The isolated [Rh(L-alaninate)(nbd)] catalyst without a cocatalyst gave a high yield (83%) and a large molecular weight (245.26 × 10^4^). This may be due to the higher stability of [Rh(L-alaninate)(cod)] and [Rh(L-alaninate)(nbd)] in solution compared to the binary [Rh(cod)Cl]_2_/(chiral amine) and [Rh(nbd)Cl]_2_/(chiral amine) catalytic system. By contrast, the isolated [Rh(L-alaninate)(cod)] and [Rh(L-alaninate)(nbd)] used as the active species directly induces the asymmetric polymerization of **DoDHPA**. However, the binary [Rh(cod)Cl]_2_/(chiral amine) and [Rh(nbd)Cl]_2_/(chiral amine) catalytic systems require coordination during the reaction so that the coordinating rate may be low. As a result, when [Rh(L-alaninate)(cod)] and [Rh(L-alaninate)(nbd)] were used, the polymerization has more active species, enabling the polymerizations of **DoDHPA** with high yields and high molecular weight.

**Figure 2:**
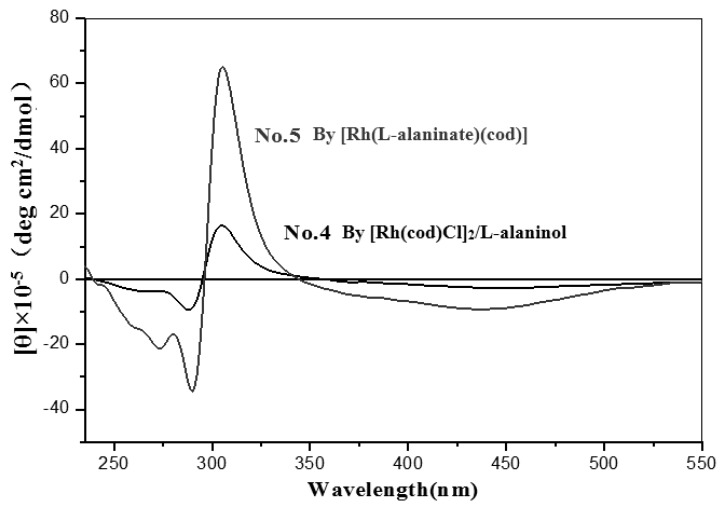
Circular dichroism (CD) spectra of chiral helical poly(**DoDHPA**) obtained using [Rh(cod)Cl]_2_/L-alaninol and [Rh(L-alaninate)(cod)] as catalysts (CD spectra were determined in THF solution with 1.000 mmol/L poly(**DoDHPA**)) (Table 1, entries 4 and 5).

The CD spectra of the polymers are shown in Figure 2. The polymers exhibited Cotton effects with molar ellipticity (θ) (deg cm^2^/dmol) at 310 and 430 nm. The peak at 310 nm may arise from a chiral position between the adjacent pendant groups [11,12]. The absorption band at 430 nm was assigned to the conjugated main chain. Therefore, the polymers had chirality in the main chain, that is, a one-handed helical backbone. The (θ) value of poly (**DoDHPA**) obtained by using the isolated [Rh(L-alaninate)(cod)] was higher (Figure 2, No. 5) than that of the polymer obtained by using the binary [Rh(cod)Cl]_2_/(L-alaninol) catalytic system (Figure 2, No. 4). When [Rh(L-alaninate)(nbd)] was used as the catalyst in CH_2_Cl_2_ solution, the obtained poly(**DoDHPA**) showed a greater molar ellipticity [θ] value than [Rh(nbd)Cl]_2_/(L-alaninol) (Figure S16, Supplementary Materials).

**Chart 1:**
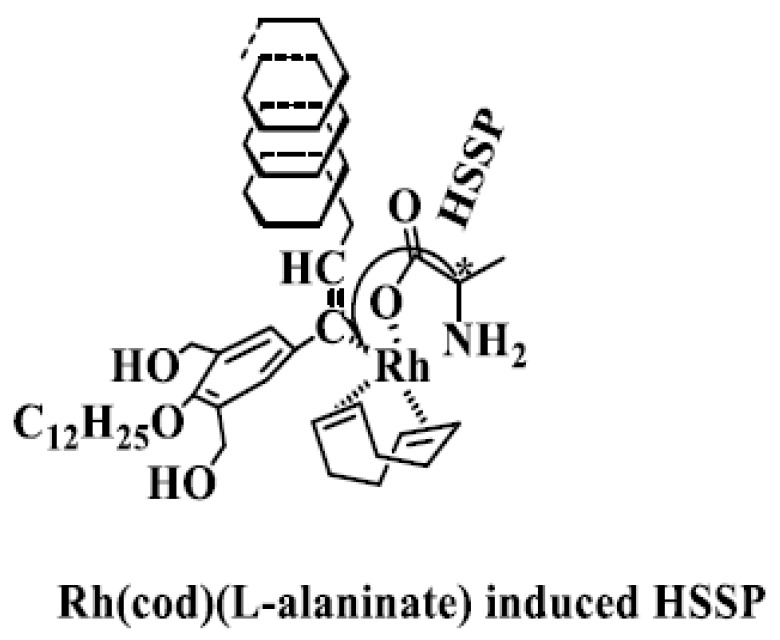
Mechanism of helix-sense-selective polymerization (**HSSP)** of **DoDHPA** by the [Rh(L-alaninate)(cod)] catalyst.

When [Rh(L-alaninate)(cod)] and [Rh(L-alaninate)(nbd)] were used as catalysts, even though the Rh-N bonds dissociate, the Rh-O bonds are preserved (Chart 1). This differences between the bonds are due to the stronger donating and acceptor abilities of the O-atom compared to the N-atom. The Rh–N bond length is greater than the Rh–O bond length, enabling proton transfer from the NH group to the coordinated monomer [17,24]. The synthesis and characterization of [Rh(L-alaninate)(cod)] and [Rh(L-alaninate)(nbd)] can confirm that the Rh complex containing the amine group may be the real active species of **HSSP** or **SHSSP** (Chart 1), providing direct evidence for our previous results [13]. Therefore, the isolated [Rh(L-alaninate)(cod)] and [Rh(L-alaninate)(nbd)] also maintains a higher helical selective ability than the binary [Rh(cod)Cl]_2_/L-alaninol and [Rh(nbd)Cl]_2_/L-alaninol catalytic system in **HSSP** of **DoDHPA.**

These findings indicate that the chiral rhodium complex with L-alaninate as the ligand is effective for **HSSP**. The [Rh(L-alaninate)(cod)] and [Rh(L-alaninate)(nbd)] catalyst can induce **HSSP** of **DoDHPA**.

**Figure 3:**
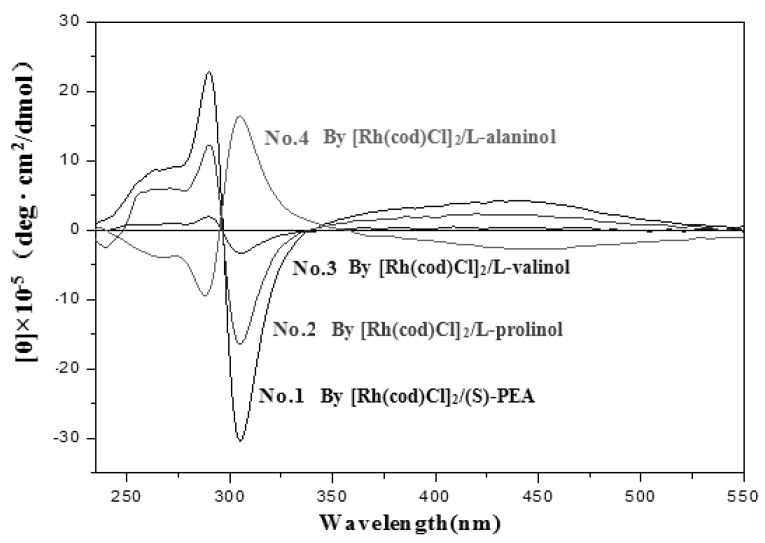
CD spectra of chiral poly(**DoDHPA**) obtained using the [Rh(cod)Cl]_2_/(chiral amine) catalytic system (CD spectra were determined in a THF solution with 1.000 mmol/L poly(**DoDHPA**)) (Table 1, entries 1–4).

On the other hand, we also investigated role of co-catalysts on **HSSP** of **DoDHPA**. As shown in Figure 3, when the binary [Rh(cod)Cl]_2_/chiral amine catalytic system was used to polymerize **DoDHPA**, the type of chiral cocatalysts affected the (θ) value of the polymers. The (θ) value of the polymer obtained by [Rh(cod)Cl]_2_/(S)-PEA was higher (Figure 3, No. 1) than those obtained by using L-prolinol, L-valinol and L-alaninol (Figure 3, Nos. 2–4). This behavior may be due to the larger steric hindrance of (S)-PEA compared to those of L-prolinol, L-valinol, and L-alaninol. These findings indicated that the (S)-PEA is a good cocatalyst in the **HSSP** of **DoDHPA**.

**Figure 4:**
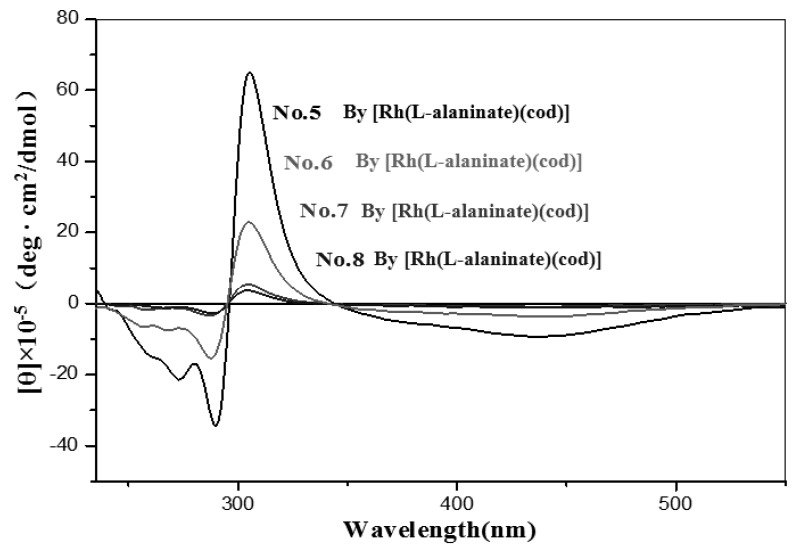
CD spectra of chiral helical poly(**DoDHPA**) obtained using the [Rh(L-alaninate)(cod)] catalyst in different solvents (CD spectra were determined in THF solution with 1.000 mmol/L poly(**DoDHPA**)) (Table 1, entries 5–8).

We also studied the effect of the solution on the **HSSP** of **DoDHPA**. When toluene was used as the polymerization solvent, [Rh(L-alaninate)(cod)] improved the catalytic performance to provide polymers with higher *M*_w_, yields and (θ) values (Table 1, No. 5 and Figure 4) compared to those obtained using CH_2_Cl_2_, benzene, and acetone as the solvents. The polarity of the solvent strongly affected the polymerization of **DoDHPA**. The polarity values of toluene and benzene are lower than those of the other solvents. **DoDHPA** and poly(**DoDHPA**) have better solubilities in toluene. It is important to mention that the solvents affect the catalytic activity. These differences may be due to the different solvents affecting the stability of the Rh complex acting as the active species of the asymmetric polymerization.

Polymerization of phenylacetylene (**PA**) [25] was also performed using a [Rh(L-alaninate)(cod)] catalyst in different solvents at 30 °C for 24 h. The obtained results are summarized in Table 2. The yield and *M*_w_ of polymers were very high, with values of 78.6–92.5% and 10^5^–3 × 10^5^, respectively. When methanol is used as the solvent, the highest yield and *M*_w_ values were obtained. This is because the methanol hydroxyl and the amino group of the active species (Chart 1) can form a hydrogen bond, improving the activity of the polymerization species and increasing the polymerization yield. Additionally the surface tension and the boiling point of methanol are low, and the poly(**PA**) solubility in methanol is strong, which can effectively reduce the viscosity of the system and promote the polymerization. Therefore, [Rh(L-alaninate)(cod)] used in a CH_3_OH solution was a high-efficiency catalyst for the polymerization of **PA**.

## 5. Conclusions

The poly(**DoDHPA**) which obtained using the chiral [Rh(L-alaninate)(cod)] and [Rh(L-alaninate)(nbd)] catalysts exhibited extremely high *M*_w_ and molar ellipticity values. The sense of the helix of poly(**DoDHPA**) was induced by the chirality sign of [Rh(L-alaninate)(cod)] and [Rh(L-alaninate)(nbd)]. Simultaneously, [Rh(L-alaninate)(cod)] and [Rh(L-alaninate)(nbd)] also exhibited high catalytic activity in phenylacetylene polymerization.

## Figures and Tables

**Table 1:** Asymmetric polymerizations of **DoDHPA** with various chiral Rh catalysts ^a^.

No.	Catalyst	Cocatalyst	Solvent	Yield	*M*_w_^b^ (× 10^4^)	*M*_w_/*M*_n_^b^	[θ]_310_^c^ (×10^−5^)
1	[Rh(cod)Cl]_2_	(S)-PEA	Toluene	23	9.82	2.35	−24.70
2		L-prolinol	Toluene	10	9.83	3.88	−13.16
3		L-valinol	Toluene	8	2.93	1.15	−2.77
4		L-alaninol	Toluene	31	28.50	5.36	13.57
5	[Rh(L-alaninate)(cod)]	none	Toluene	72	55.43	2.14	55.00
6		none	CH_2_Cl_2_	25	4.57	2.30	19.10
7		none	Benzene	39	15.14	2.64	4.26
8		none	Acetone	17	20.64	3.95	2.83
9 [12]	[Rh(nbd)Cl]_2_	(R)-PEA	Toluene	36	517.00	-	31.10
10		L-alaninol	Toluene	64	43.39	2.78	29.00
11		L-alaninol	CH_2_Cl_2_	35	20.65	4.93	17.00
12	[Rh(L-alaninate)(nbd)]	none	Toluene	33	73.08	2.83	6.27
13		none	CH_2_Cl_2_	83	245.26	5.25	79.11

^a^*A*_t_ 35 °C, 24 h; [monomer]=0.100 mol/l, [monomer]/[catalyst]=100, [cocatalyst]/[catalyst] = 250.^b^ Determined by GPC correlating polystyrene standard with THF eluent.^c^ Molar ellipticity [θ] (deg cm^2^/dmol) at 310 nm from CD spectra, 1.000 mmol/L, in THF.

**Table 2:** Polymerizations of phenylacetylene with [Rh(L-alaninate)(cod)] as the catalyst ^a^.

No.	Solvent	Yield(%)	*M*_n_^b^(×10^4^)	*M*_w_^b^ (×10^4^)	*M*_w_/*M*_n_^b^
1	Toluene	78.6	6.80	10.54	1.55
2	CHCl_3_	82.5	9.58	12.74	1.33
3	CH_2_Cl_2_	82.0	10.32	21.57	2.09
4	THF	87.8	12.78	28.49	2.23
5	CH_3_OH	92.5	17.26	30.21	1.75

^a^*A*_t_ 30 °C, 24 h; [phenylacetylene] = 1.0 mmol, [Rh] = 0.010 mmol. ^b^ Determined by GPC correlating polystyrene standard with a THF eluent.

## Supplementary Materials

The following are available online at http://www.mdpi.com/2073-4360/10/11/1223/s1, Chart S1: Chemical Structures of The Monomers (**ORDHPAs**), Scheme S1: Synthetic route to catalyst [Rh(L-alaninate)(nbd)], Figure S1: ^13^C NMR spectrum of [Rh(cod)Cl_2_] in CDCl_3_ at 25 °C, Figure S2: FT-IR spectrum of [Rh(cod)Cl_2_]. Figure S3: ^13^C NMR spectrum of [Rh(L-alaninate)(cod)] in DMSO-*d*_6_ at 25 °C, Figure S4: FT-IR spectrum of [Rh(L-alaninate)(cod)], Figure S5: ^1^H NMR spectrum of [Rh(L-alaninate)(nbd)] in DMSO-*d*_6_ at 25 °C, Figure S6: FT-IR spectrum of [Rh(L-alaninate)(nbd)], Figure S7: ^1^H NMR spectrum of achiral monomer **DoDHPA** in CDCl_3_ at 25 °C, Figure S8: FT-IR spectrum of achiral monomer **DoDHPA**, Figure S9: ^1^H NMR spectrum of poly(**DoDHPA**) in 1,2-dichlorobenzene-*d*_4_, Figure S10: FT-IR spectrum of poly(**DoDHPA**), Figure S11: SEM of the [Rh(L-alaninate)(cod)], Figure S12: XRD spectrum of the [Rh(L-alaninate)(cod)], Figure S13: Mass spectrum of the [Rh(L-alaninate)(cod)], Figure S14: XRD spectrum of the [Rh(L-alaninate)(nbd)], Figure S15: SEM of the [Rh(L-alaninate)(nbd)], Figure S16: CD spectra of chiral poly(**DoDHPA**) obtained using the [Rh(nbd)Cl]_2_/(L-alaninol) and [Rh(L-alaninate)(nbd)] catalyst in different solvents (CD spectra were determined in a THF solution with 1.000 mmol/L poly(**DoDHPA**)) (Table 1, entries 10–13).
