# Supplementary material for: [Rh(L-alaninate)(1,5-Cyclooctadiene)] Catalyzed Helix-Sense-Selective Polymerizations of Achiral Phenylacetylenes
Source: Polymers (Basel). 2018 Nov 3;10(11):1223. doi: 10.3390/polym10111223 (PMC6290612; doi:10.3390/polym10111223)
Supplement: Supplementary file 1 [file polymers-10-01223-s001.pdf]

polymers

Polymers 2018

OPEN ACCESS

polymers

ISSN 2073-4360

www.mdpi.com/journal/polymers

Article

Supporting Information

Rh(L-alaninate)(1,5-Cyclooctadiene) catalyzed helix-sense-selective polymerizations of achiral phenylacetylenes

Qingyu Wang [1], Hongge Jia [1,*], Yongqiang Shi [2,*], Liqun Ma [1], Guoxing Yang [3], Yazhen Wang [1], Shuangping Xu [1], Jianjun Wang [1], Yu Zang [1], Toshiki Aoki [1,4]

[1] College of Materials Science and Engineering, College of Chemistry and Chemical Engineering , Heilongjiang Province Key Laboratory of Polymeric Composition Material, Qiqihar University, Wenhua Street 42, Qiqihar, 161006, China; 810681496@qq.com (Q.W.); maliqun6166@163.com (L.M.); wyz6166@163.com (Y.W.); xshp_1979_1999@163.com (S.X.); wangjianjun860505@163.com (J.W.); zangyu25@163.com (Y.Z.)

[2] Southern University of Science and Technology, Xilixueyuan Street 1088, Shenzhen, 518055, China;

[3] Daqing Petrochemical Research Center, Petrochemical Research Institute, China National Petroleum Corporation, Chengxiang Road 2, Daqing 163714, People's Republic of China. ygx459@petrochina.com.cn

[4] Graduate School of Science and Technology, Niigata University, 2-8050 Ikarashi, Nishi-ku, Niigata 950-2181, Japan; prof.aoki@gmail.com

* Corresponding author: Hongge Jia (Tel.: +86-452-2738752; E-mail address: jiahongge11@hotmail.com); Yongqiang Shi (Tel.: +86-18345293620; E-mail address: shiyq@sustu.edu.cn).

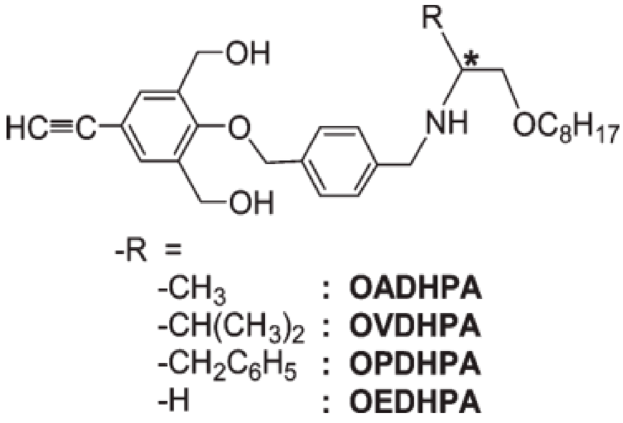

Chart S1. Chemical Structures of the Monomers (**ORDHPAs**)

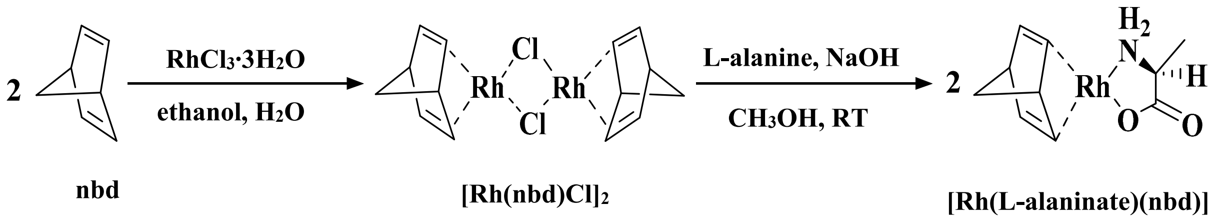

Scheme S1. Synthetic route to catalyst [Rh(L-alaninate)(nbd)].

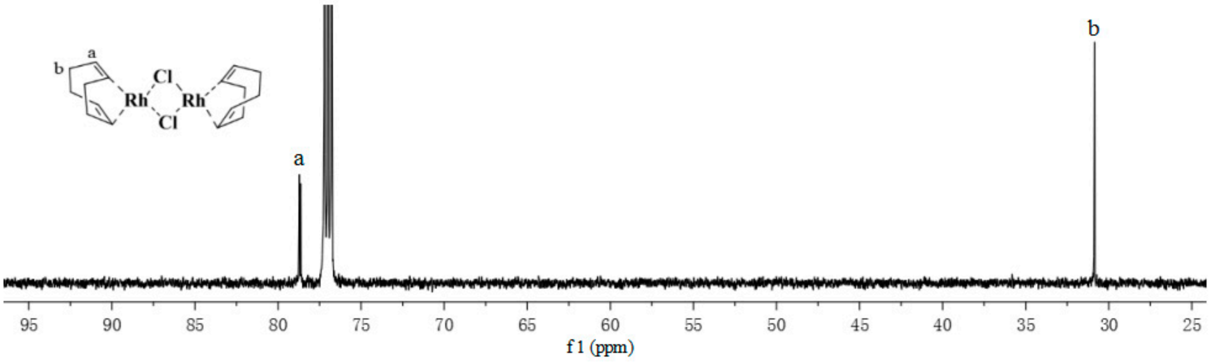

Figure S1. ^{13}C-NMR spectrum of [Rh(cod)Cl$_2$] in $CDCl_3$ at 25 °C.

^{13}C-NMR (150 MHz, $CDCl_3$, TMS, δ): 78.63 (*C*H=CH), 30.85 (CH*C*H$_2$*C*H$_2$).

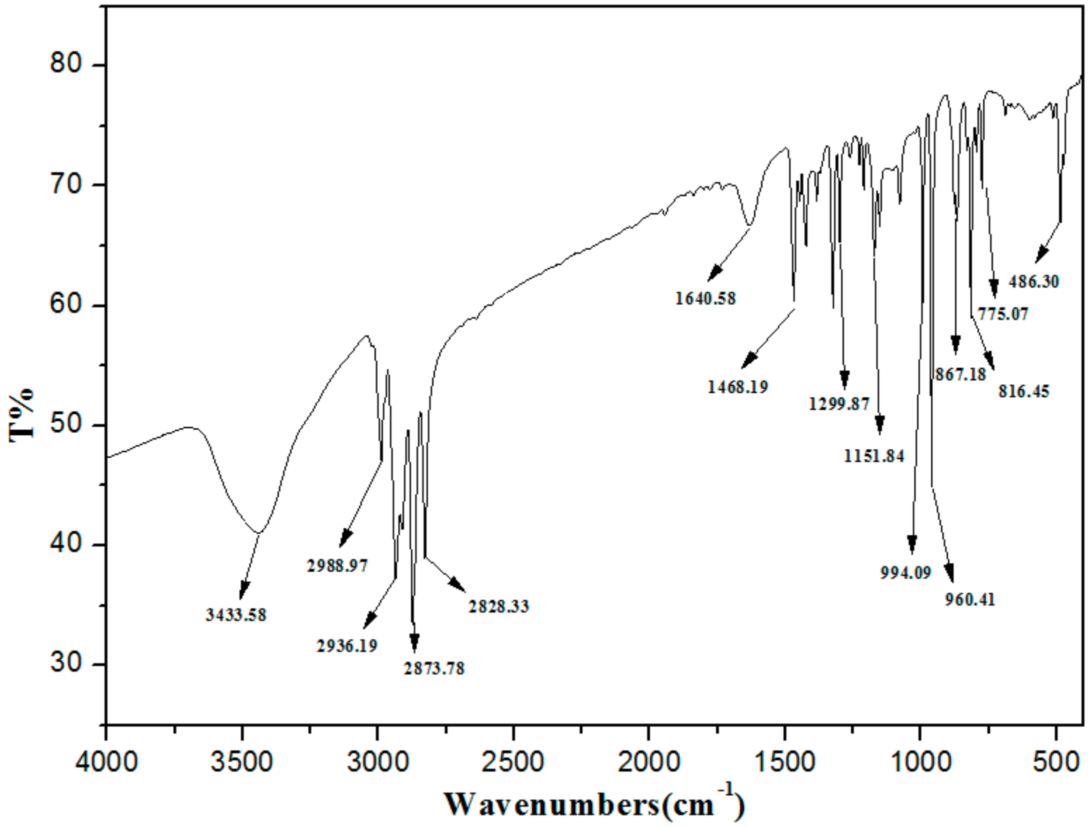

Figure S2. FT-IR spectrum of [Rh(cod)Cl_2].

IR (KBr): 3433.58, 2988.97, 2936.19, 2873.78, 2828.33, 1640.58, 1468.19, 1299.87, 1151.84, 994.09, 960.41, 867.18, 816.45, 775.07, 486.30 cm^{-1}.

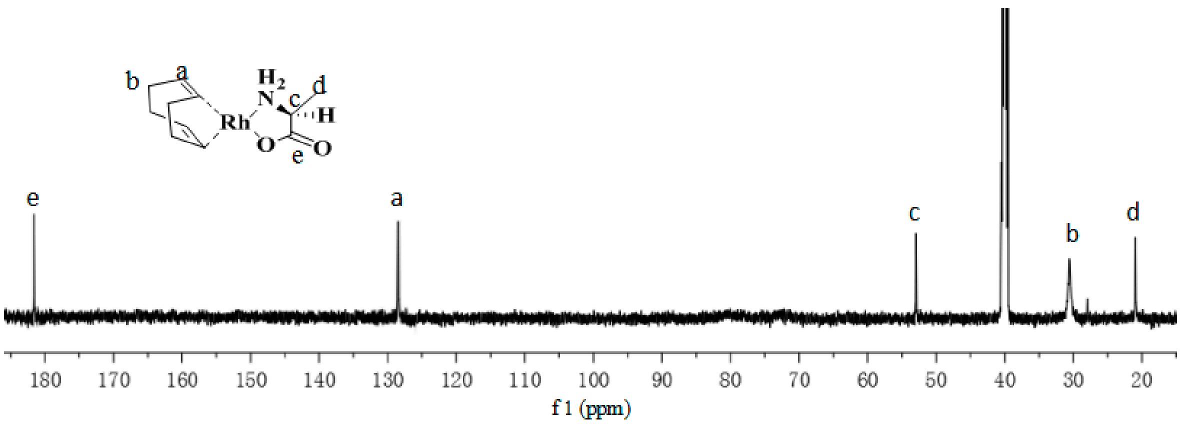

Figure S3. ^{13}C-NMR spectrum of [Rh(L-alaninate)(cod)] in DMSO-d_6 at 25 °C.

^{13}C-NMR (150 MHz, DMSO-d_6, TMS, δ): 183.70 (*C*OO), 129.04 (*C*H=CH), 52.928 (CH_3*C*H), 30.60 (*C*$H_2$$CH_2$), 20.92 (*C*$H_3$CH).

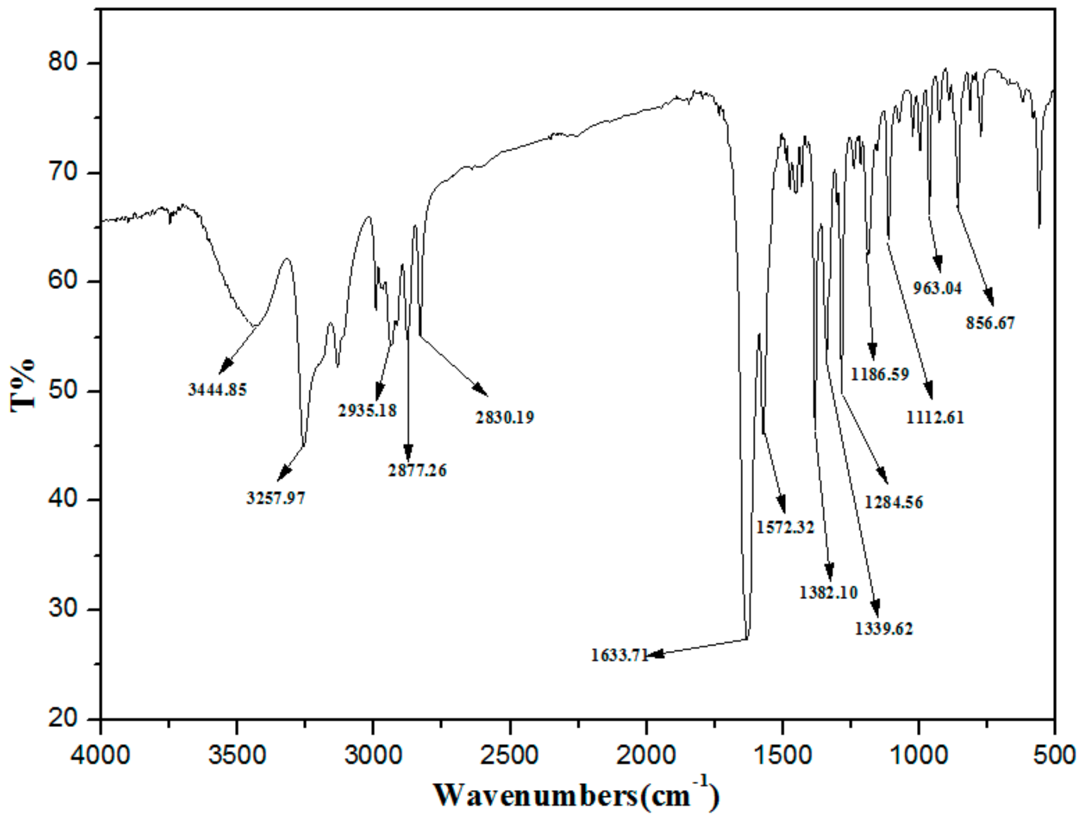

Figure S4. FT-IR spectrum of [Rh(L-alaninate)(cod)].

IR (KBr): 3444.85, 3257.97, 2935.18, 2877.26, 2830.19, 1633.71, 1572.32, 1382.10, 1339.62, 1284.56, 1186.59, 1112.61, 963.04, 856.67 cm^{-1}.

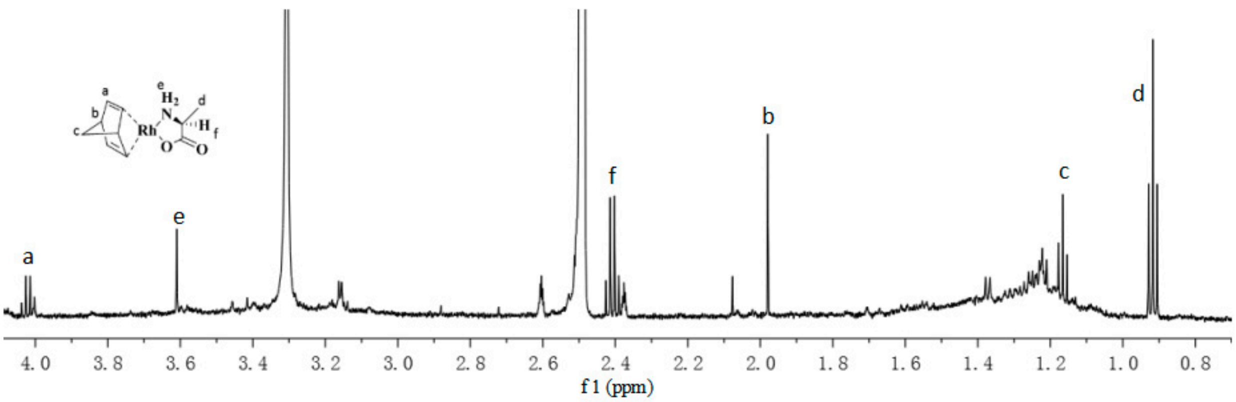

Figure S5. ^{1}H-NMR spectrum of [Rh(L-alaninate)(nbd)] in DMSO-d_6 at 25 °C.

^{1}H-NMR (600 MHz, DMSO-d_6, TMS, δ): 4.02 (m, 4H, CH=C*H*), 3.61 (s, 2H, $CH_3CHN\mathit{H_2}$), 2.40 (m, 1H, $CH_3C\mathit{H}NH_2$), 1.98 (s, 2H, $C\mathit{H}CH_2CH$), 1.17 (t, 2H, $CHC\mathit{H_2}CH$), 0.92 (t, 3H, $C\mathit{H_3}CHNH_2$).

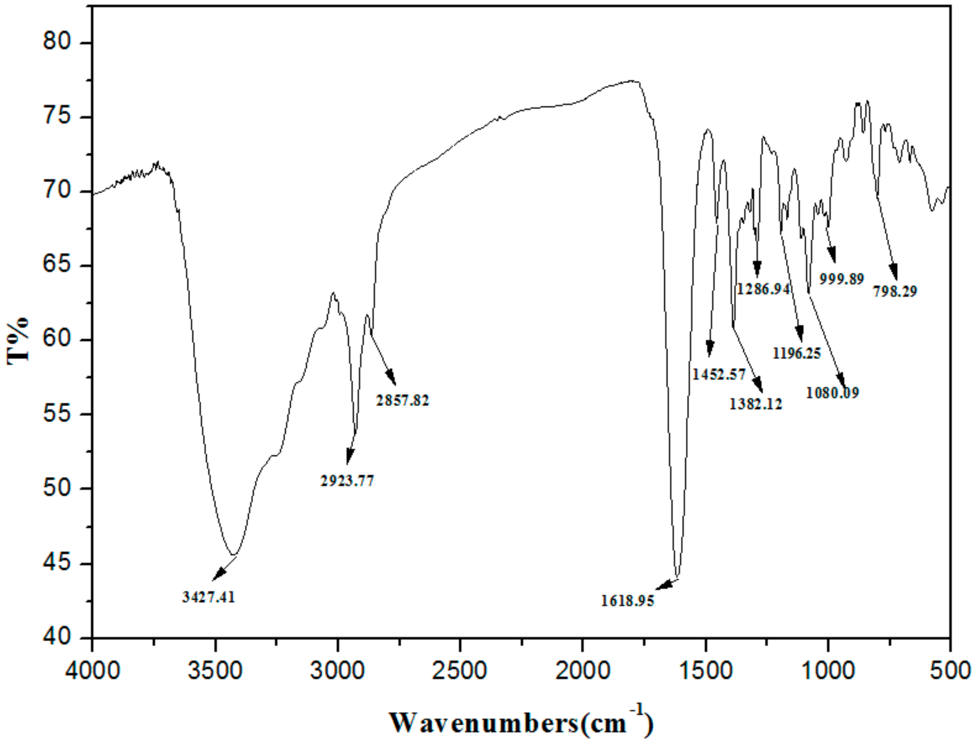

Figure S6. FT-IR spectrum of [Rh(L-alaninate)(nbd)].

IR (KBr): 3427.41, 2923.77, 2857.82, 1618.95, 1452.57, 1382.12, 1286.94, 1196.25, 1080.09, 999.89, 798.29 cm^{-1}.

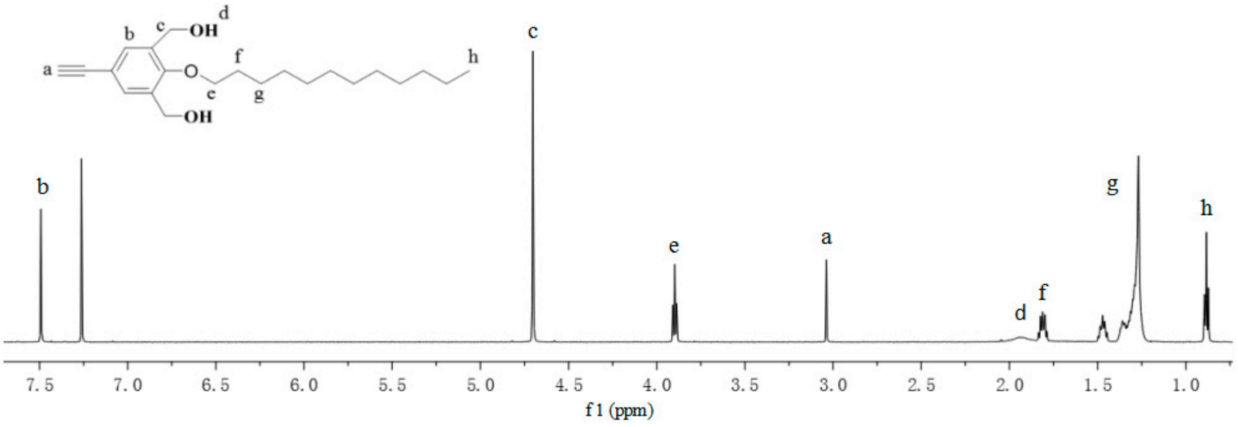

Figure S7. ^{1}H-NMR spectrum of achiral monomer **DoDHPA** in $CDCl_3$ at 25 °C.

^{1}H-NMR (600 MHz, $CDCl_3$, TMS, δ): 7.49 (s, 2H, Ph*H*), 4.70 (s, 4H, C*H*$_2$OH), 3.88 (t, 2H, OC*H*$_2$CH$_2$), 3.05 (s, 1H, C≡C*H*), 1.93 (br, 2H, CH$_2$O*H*), 1.78 (m, 2H, OCH$_2$C*H*$_2$CH$_2$), 1.26-1.48 (br, 18H, OC$_2$H$_4$(C*H*$_2$)$_9$CH$_3$), 0.88 (t, 3H, C*H*$_3$).

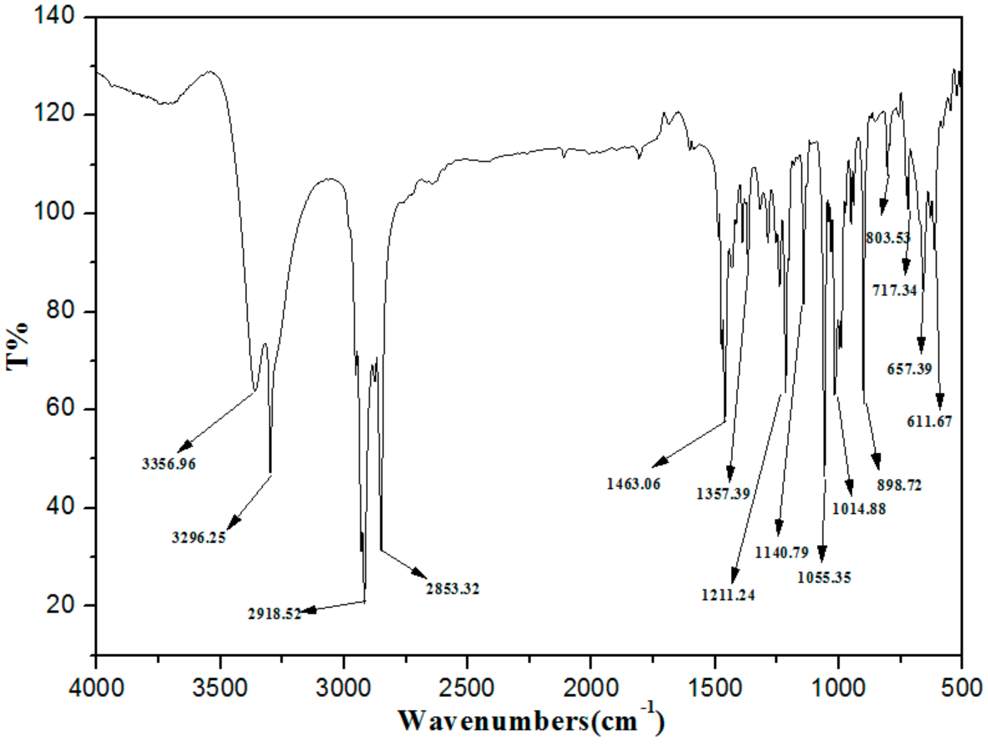

Figure S8. FT-IR spectrum of achiral monomer **DoDHPA**.

IR (KBr): 3356.96, 3296.25, 2918.52, 2853.32, 1463.06, 1357.39, 1211.24, 1140.79, 1055.35, 1014.88, 898.72, 803.53, 717.34, 657.39, 611.67 cm^{-1} .

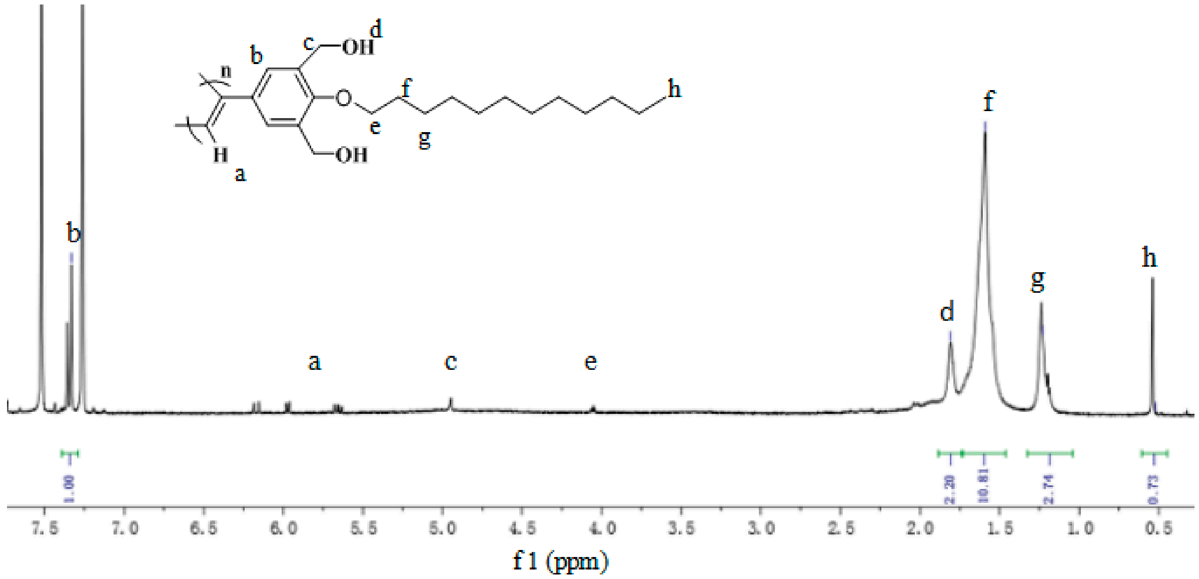

Figure S9. ^{1}H-NMR spectrum of poly(**DoDHPA**) in 1,2-dichlorobenzene-d_4.

^{1}H-NMR (600 MHz, 1,2-dichlorobenzene-d_4, TMS, δ): 7.33 (s, 2H, Ph*H*), 6.18-5.63 (m, 1H, PhC=C*H*), 4.95 (d, 4H, CH_2OH), 4.06 (t, 2H, OCH_2CH_2), 1.77 (t, 2H, CH_2O*H*), 1.59 (s, 2H, OCH_2CH_2CH_2), 1.11-1.23 (m, 18H, OC_2H_4(CH_2)$_9$CH_3), 0.53 (t, 3H, CH_3).

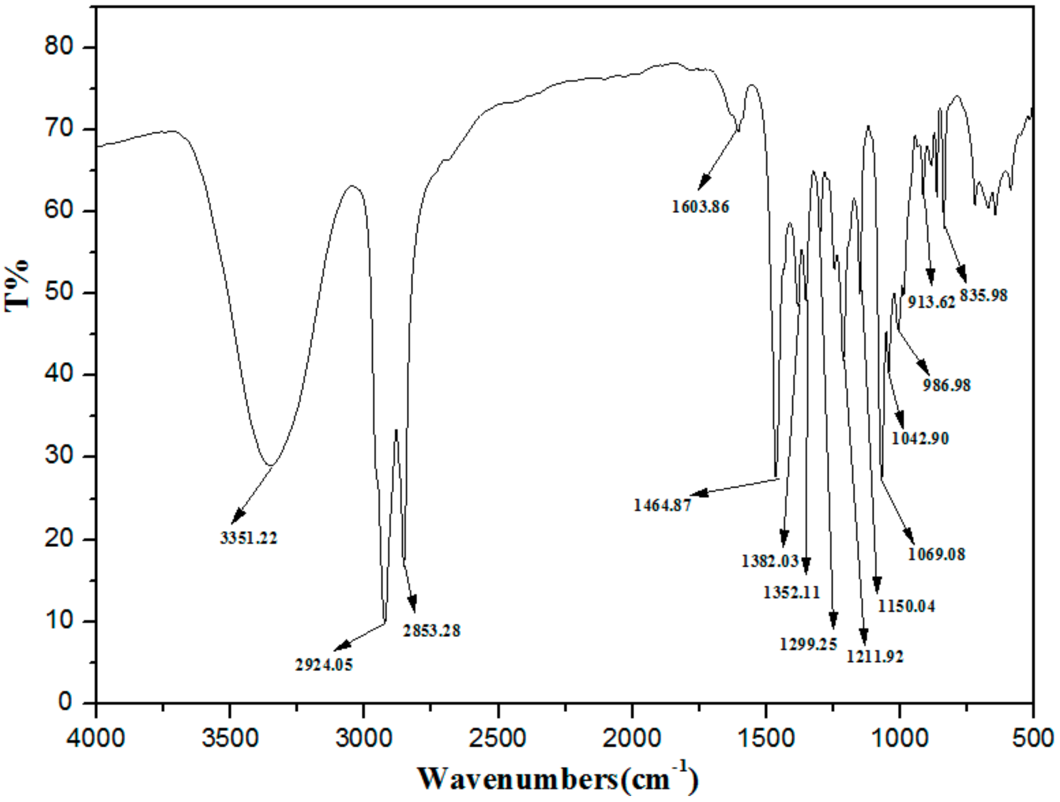

Figure S10. FT-IR spectrum of poly(**DoDHPA**).

IR (KBr): 3351.22, 2924.05, 2853.28, 1603.86, 1464.87, 1382.03, 1352.11, 1299.25, 1211.92, 1150.04, 1069.08, 1042.90, 986.98, 913.62, 835.98 cm^{-1}.

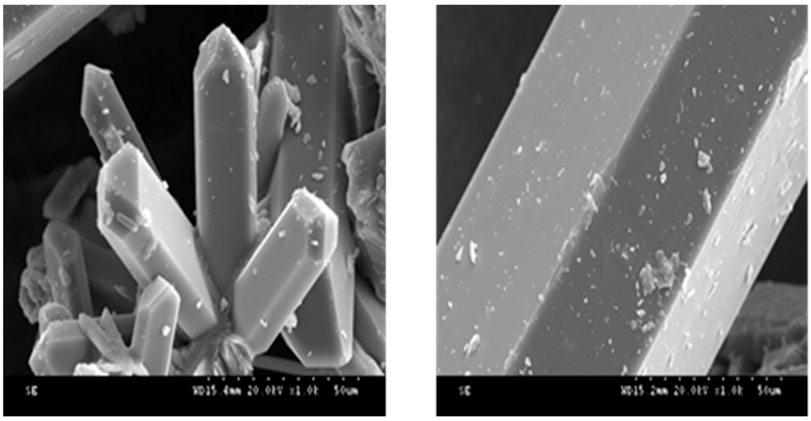

Figure S11. SEM of the [Rh(L-alaninate)(cod)].

SEM micrographs were measured by a Hitachi S4300 electron microscope (Hitachi, Ltd, Japan) with a 20 kV accelerating voltage.

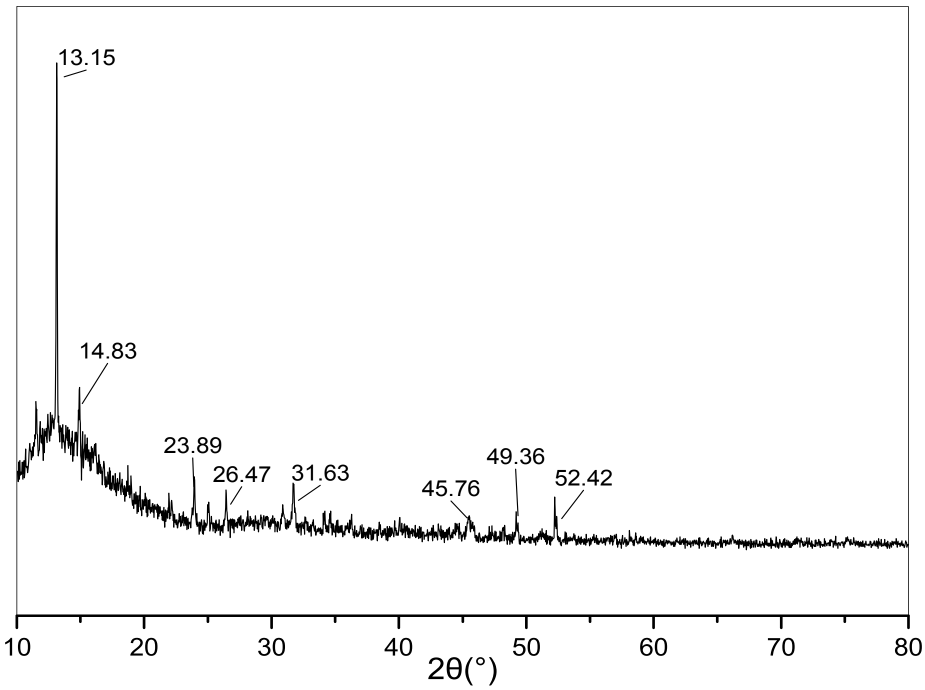

Figure S12. XRD spectrum of the [Rh(L-alaninate)(cod)].

The XRD spectrum showed that the [Rh(L-alaninate)(cod)] has peaks at 13.15°, 14.83°, 23.89°, 26.47°, 31.63°, 45.76°, 49.36° and 52.42°, corresponding to the crystal plane spacing d values of 6.68 nm, 5.96 nm, 3.72 nm, 3.36 nm, 2.85 nm, 1.98 nm, 1.84 nm and 1.74 nm, respectively.

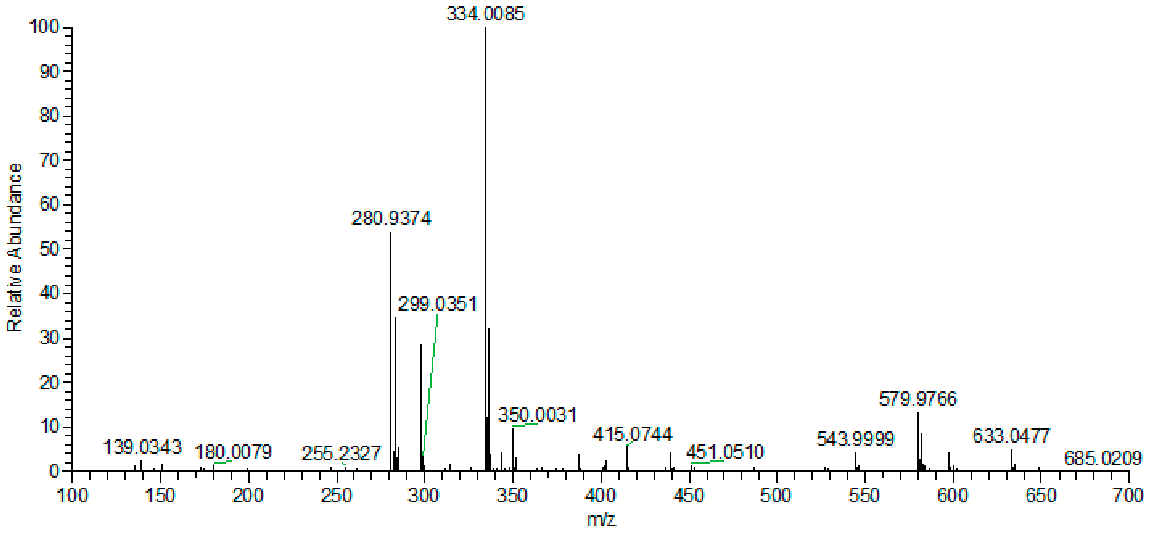

Figure S13. Mass spectrum of the [Rh(L-alaninate)(cod)].

Mass spectra were recorded using a UltiMate 3000 UPLC/Q-Exactive Orbitrap MS (Thermo, America) instrument.

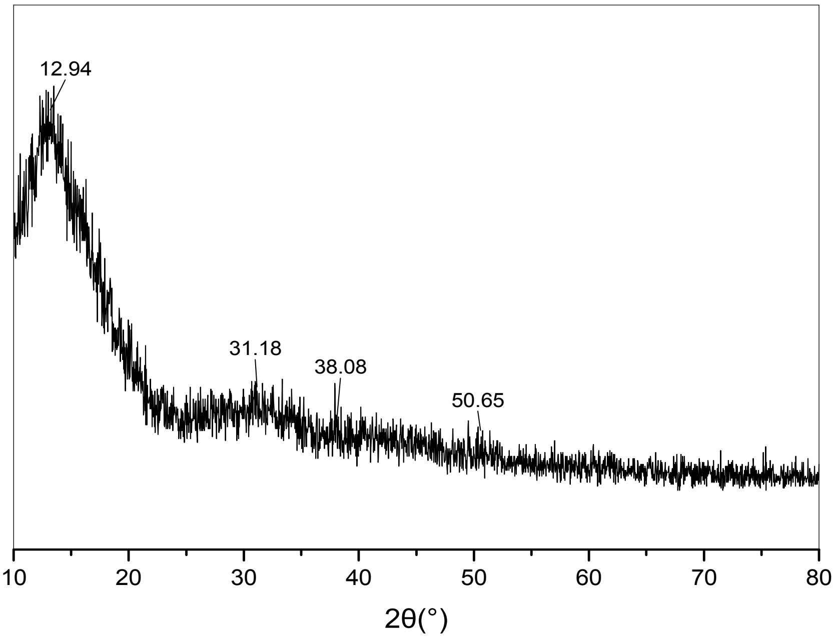

Figure S14. XRD spectrum of the [Rh(L-alaninate)(nbd)].

The XRD spectrum showed that the [Rh(L-alaninate)(nbd)] appear different peaks at 12.94°, 31.18°, 38.08° and 50.65°, corresponding crystal plane spacing d values are 6.83 nm, 2.86 nm, 2.36 nm, and 1.79 nm respectively.

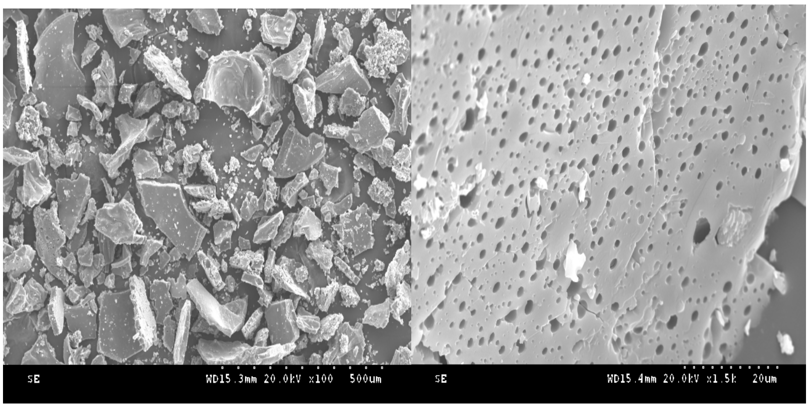

Figure S15. SEM of the [Rh(L-alaninate)(nbd)].

SEM micrographs were measured by a Hitachi S4300 electron microscope (Hitachi, Ltd, Japan) with a 20 kV accelerating voltage.

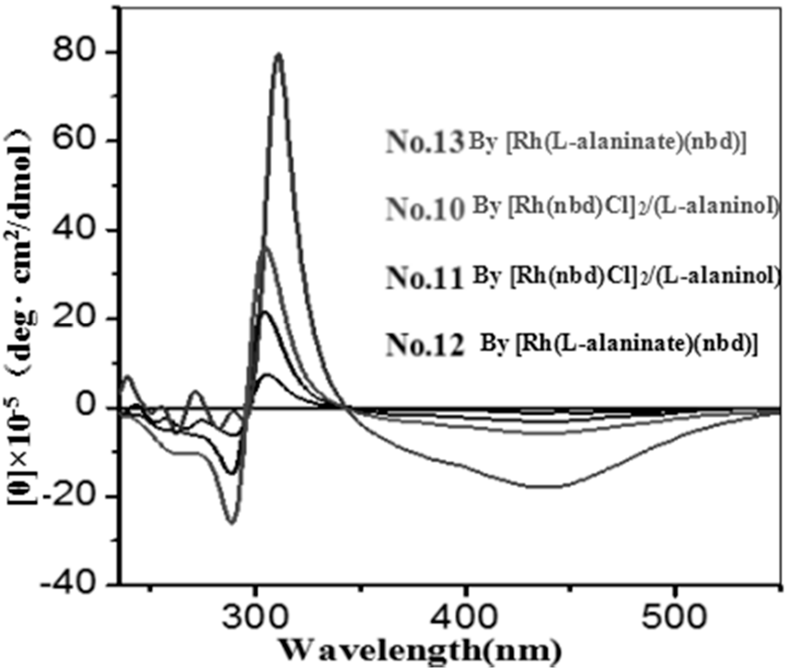

Figure S16. CD spectra of chiral poly(**DoDHPA**) obtained using the $[Rh(nbd)Cl]_2$/(L-alaninol) and [Rh(L-alaninate)(nbd)] catalyst in different solvents (CD spectra were determined in a THF solution with 1.000 mmol/L poly(**DoDHPA**)) (Table 1, entries 10-13)
